# MuralsNet: Dunhuang Decorative Mural Image Inpainting Based on BiFormer and GLM Fusion

**DOI:** 10.3390/s26185707

**Published:** 2026-09-09

**Authors:** Hui Ren, Wenjing Yue, Chengya Zhang, Zhibin Su

**Affiliations:** 1 School of Information and Communication Engineering, Communication University of China, Beijing 100024, China; renhui@cuc.edu.cn (H.R.); y18939297931@163.com (W.Y.); zcy18734423773@163.com (C.Z.); 2 Key Laboratory of Acoustic Visual Technology and Intelligent Control System, Ministry of Culture and Tourism, Communication University of China, Beijing 100024, China; 3 State Key Laboratory of Media Convergence and Communication, Communication University of China, Beijing 100024, China

**Keywords:** Dunhuang murals, image inpainting, EdgeConnect, BiFormer, GLM

## Abstract

**Highlights:**

**What are the main findings?**

- A structure-guided global–local collaborative framework, termed MuralsNet, is proposed for restoring complex decorative patterns in Dunhuang mural images.
- MuralsNet achieves the best PSNR and SSIM among the evaluated methods, while showing better preservation of fine decorative structures in the visual comparisons.

**What are the implications of the main findings?**

- The proposed method provides an automated restoration approach for preserving complex decorative structures in Dunhuang murals.
- The framework offers a general reference for applying deep learning-based image restoration to other cultural heritage preservation tasks.

**Abstract:**

Diverse costume and decorative patterns in Dunhuang murals have significant historical and cultural value. However, natural erosion and material aging have caused cracks, flaking, and missing regions. Existing image restoration methods are still challenged by complex decorative patterns, including insufficient modeling of long-range structural dependencies, incoherent local textures, and weak global consistency. To address these problems, this paper proposes MuralsNet, an EdgeConnect-based inpainting framework adapted to the structural characteristics of Dunhuang decorative murals. The original two-stage strategy of EdgeConnect (G1, G2) is retained to provide explicit edge guidance, while two targeted modifications are introduced into the G2 image completion network. First, BiFormer is embedded into the bottleneck stage of G2 U-Net to capture global contextual information and long-range dependencies through a bi-level routing attention mechanism. Meanwhile, a Global–Local Fusion Module (GLM) is constructed in the decoder stage to facilitate the effective fusion of high-level semantic features and local detail features, thereby improving structural coherence and texture restoration in damaged regions. Experiments show that MuralsNet achieves the highest PSNR, SSIM, and Masked Edge F1, as well as the lowest LPIPS, among the evaluated methods, while LaMa obtains the lowest MAE. Visual comparisons further show that MuralsNet better preserves fine decorative contours and local structural details in several representative examples. Ablation experiments further show that BiFormer and the GLM provide complementary improvements within the proposed framework. These results demonstrate the potential of the proposed task-oriented integration strategy for the digital restoration of complex decorative mural images.

## 1. Introduction

Image inpainting aims to reconstruct missing content while preserving structural consistency, texture continuity, and visual fidelity [1,2]. Traditional image restoration methods have also been formulated using variational models, which provide mathematically interpretable priors for reconstructing degraded image structures. Recent studies have further introduced fractional-order operators to enhance the modeling of nonlocal information and structural details. For example, Lian et al. developed a fractional Laplacian-based variational model for image inpainting [3], while Liu et al. extended fractional-order variational modeling to nighttime image dehazing [4]. Although these methods provide effective mathematical priors for image restoration, reconstructing large irregular missing regions with complex semantic and decorative structures remains challenging. With the rapid development of deep learning, image inpainting methods have been widely applied in various scenarios, including image completion, object removal, and digital heritage preservation [5]. However, compared with natural images, cultural heritage images exhibit unique challenges for image inpainting due to their complex structural patterns, repeated decorative elements, and irregular degradation characteristics. In particular, mural images often contain continuous curves, symmetric layouts, fine textures, and highly organized color distributions, making it difficult for existing restoration methods to simultaneously maintain global structural coherence and local detail consistency. Among various cultural heritage images, Dunhuang murals provide a representative and challenging restoration scenario because their decorative patterns contain rich historical information and complex visual structures [6]. Nevertheless, long-term environmental erosion, material aging, and human activities have resulted in cracks, pigment fading, stains, and missing regions, which severely affect the integrity of decorative patterns and overall visual appearance. Therefore, digital image inpainting can provide a useful technical means for assisting the preservation and visual reconstruction of damaged Dunhuang mural images.

From a technical perspective, Dunhuang decorative mural inpainting is not merely a pixel-filling task, but a challenging problem involving structural reconstruction, pattern reasoning, and texture refinement. Specifically, three major challenges should be considered during the restoration process. First, decorative elements such as costumes, ornaments, and border patterns often contain repeated structures, symmetric layouts, directional strokes, and continuous curves. Information required to reconstruct a missing region may therefore be distributed across spatially distant regions, making long-range dependency modeling important for maintaining pattern consistency. Second, color boundaries, line contours, and fine textures are highly coupled in mural images. Inaccurate local restoration may introduce edge discontinuities, texture inconsistencies, or abrupt color transitions, reducing the visual coherence of the restored results. Third, real-world mural degradation usually presents irregular missing regions [7], where the original content cannot be directly inferred from neighboring pixels. Consequently, explicit structural guidance is useful for decorative murals because the continuity of contours and pattern boundaries strongly influences the perceived integrity of the restored result.

To address these technical challenges, existing image inpainting methods have been developed from different perspectives. Early structure-guided methods utilize explicit structural priors to guide missing-region recovery [8]. Edge-guided frameworks further improve structural consistency through predicted contour information [9]. Although these methods can preserve basic contours and boundaries, they are still limited in modeling complex long-range relationships among repeated decorative patterns. With the development of deep learning, attention-based and feature fusion methods have been explored for mural restoration by enhancing semantic interaction and local detail reconstruction [10,11,12]. Transformer-based mural restoration further strengthens long-range contextual modeling [13]. Mask-aware attention mechanisms have also been developed to improve the restoration of large and irregular missing regions [14]. Large receptive field convolution-based methods further improve structural completion by exploiting global context [15]. Recent studies have explored semantic information utilization and enhancement in different task-oriented scenarios [16,17], while semantic feedback and iterative correction have also been introduced to progressively refine image inpainting results [18]. However, without explicit structural guidance, these methods may still encounter difficulties in maintaining precise boundaries and fine-grained texture continuity. Meanwhile, adversarial learning-based image inpainting methods have also demonstrated strong capability in synthesizing realistic textures and completing complex missing regions [19]. Diffusion-based mural restoration further improves visual realism and generative flexibility [20,21]. Nevertheless, the stochastic generation process may introduce content deviations, which is undesirable for cultural heritage images requiring strict preservation of original decorative structures. Overall, existing approaches have achieved significant progress in image inpainting; however, effectively balancing structural preservation, global semantic consistency, and local texture refinement remains challenging for Dunhuang decorative mural restoration.

Consequently, the major challenge in Dunhuang decorative mural restoration lies in effectively integrating structural guidance, global reasoning, and local refinement within a unified framework rather than relying on a single restoration capability. Structural guidance provides explicit contour constraints for damaged regions, global reasoning captures long-range dependencies among repeated patterns and symmetric structures, and local refinement enhances the consistency of restored boundaries, textures, and color transitions. However, existing methods usually emphasize one specific aspect while lacking effective collaboration among these complementary abilities. As a result, structural discontinuity, unnatural texture fusion, and insufficient visual consistency may still occur when restoring complex decorative patterns, large irregular missing regions, and high-frequency boundary areas. Therefore, developing a unified framework that jointly models structure, global context, and local details is crucial for achieving high-quality Dunhuang decorative mural image inpainting, especially for decorative mural images with highly repetitive and structured patterns.

Motivated by these observations, we propose MuralsNet, a structure-guided global–local collaborative framework for Dunhuang decorative mural image inpainting. MuralsNet is built upon the two-stage architecture of EdgeConnect [9], in which edge prediction provides explicit structural guidance for subsequent image completion. To improve the restoration of complex decorative patterns, BiFormer is introduced into the bottleneck of the image completion network to capture long-range dependencies among spatially separated regions, while GLM is employed in the decoder to enhance the interaction between semantic information and local detail features. By operating at different stages of feature reconstruction, the two components complement the structural guidance provided by EdgeConnect and improve the representation of both global pattern relationships and local restoration details.

The main contributions of this paper are as follows:

1. A structure-guided global–local collaborative framework, MuralsNet, is developed for Dunhuang decorative mural image inpainting. Based on the two-stage restoration strategy of EdgeConnect, the framework combines explicit structural guidance with global contextual modeling and local feature refinement to address the restoration of complex decorative patterns.
2. BiFormer is introduced into the bottleneck stage of the G2 network to capture long-range dependencies among repeated patterns and spatially separated decorative structures, improving the utilization of global contextual information during image completion.
3. A Global–Local Fusion Module (GLM) is incorporated into the decoder to enhance the interaction between high-level semantic information and local detail features, with particular attention to directional structures, texture transitions, and pattern boundaries.

## 2. Dataset Construction

### 2.1. Dataset of Dunhuang Decorative Mural Images

A dataset for Dunhuang decorative mural image inpainting was constructed based on The Complete Collection of Dunhuang Murals. The selected images mainly focus on decorative regions, including costume and ornament patterns, architectural decorations, utensil motifs, plant elements, and border patterns. These regions contain rich structural patterns, repeated decorative elements, and diverse texture characteristics, providing representative samples for evaluating mural image restoration methods. To enable supervised training and quantitative evaluation, the selected samples were required to contain sufficiently complete decorative content in the regions used for cropping, so that the original cropped images could serve as reference images for simulated damage generation.

The original mural images were obtained from high-resolution digital scans and processed through manual selection and preprocessing. Specifically, all images were cropped into fixed-size patches and resized to 256 × 256 pixels. Color normalization and data augmentation were subsequently applied to improve the diversity of training samples. A unified naming and indexing strategy was adopted to facilitate dataset management and experimental reproduction.

To avoid data leakage caused by highly similar cropped regions, this study adopts a source-level data partition strategy. Specifically, original mural images were first divided into training and testing sets, and image cropping was performed after the split operation. Therefore, patches generated from the same original mural image would not appear simultaneously in both training and testing sets.

The final dataset contains 55,562 decorative mural images, including 44,384 images for training, 5589 images for validation, and 5589 images for testing. During training, data augmentation and mask generation were performed only on the training set. The validation set was used for model selection and hyperparameter adjustment without participating in gradient-based optimization, while the testing set was exclusively used for final performance evaluation and was not involved in model optimization.

### 2.2. Masked Dataset

**Figure 1:**
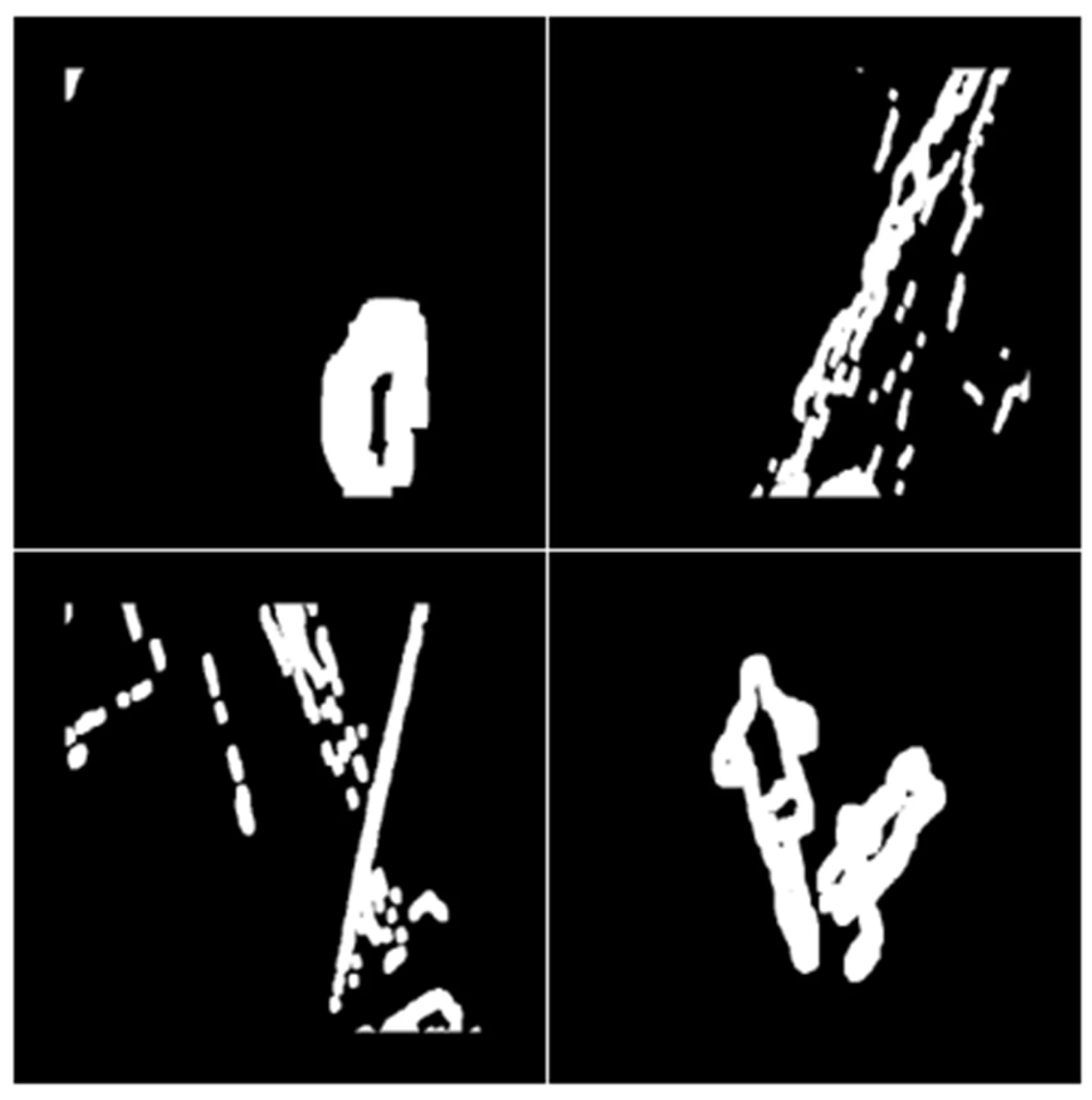
Representative irregular masks from the NVIDIA Irregular Mask Dataset.

To enhance the model’s ability to handle complex occlusions and missing regions, this study uses the random irregular dataset released by NVIDIA [22] as missing-region templates, with a total of 12,000 mask images, as shown in Figure 1.

During data preparation, irregular masks were applied to the original mural patches to generate simulated damaged inputs, while the unmasked images were retained as ground truth for quantitative evaluation. These masks provide diverse missing-region geometries and are suitable for evaluating image completion under controlled conditions. However, they primarily simulate spatial content loss rather than the full physical degradation process of real murals; therefore, more complex real-world deterioration will be considered in future work.

## 3. Digital Image Restoration Methods

### 3.1. Basic Framework and Improvement Ideas

MuralsNet is a structure-guided global–local collaborative restoration framework. It adopts EdgeConnect as the basic framework, using its two-stage restoration strategy of “structure first, content next” to provide structural priors, and further introduces targeted improvements for global modeling and local detail recovery. EdgeConnect divides the image inpainting process into a structure prediction stage (G1) and an image restoration stage (G2) [9]. In the structure prediction stage, the network extracts and analyzes input image features, learns edge information in the missing regions, and generates an estimated edge map for the missing region. In the image restoration stage, the original damaged image and the predicted edge map from the structure prediction stage are jointly fed into the network, and the texture, color, and semantic content of the missing regions are recovered through an encoder–decoder reconstruction process. With the guidance of edge information, the network can alleviate blurring and distortion caused by direct completion to some extent, thereby providing explicit structural guidance for subsequent image completion.

**Figure 2:**
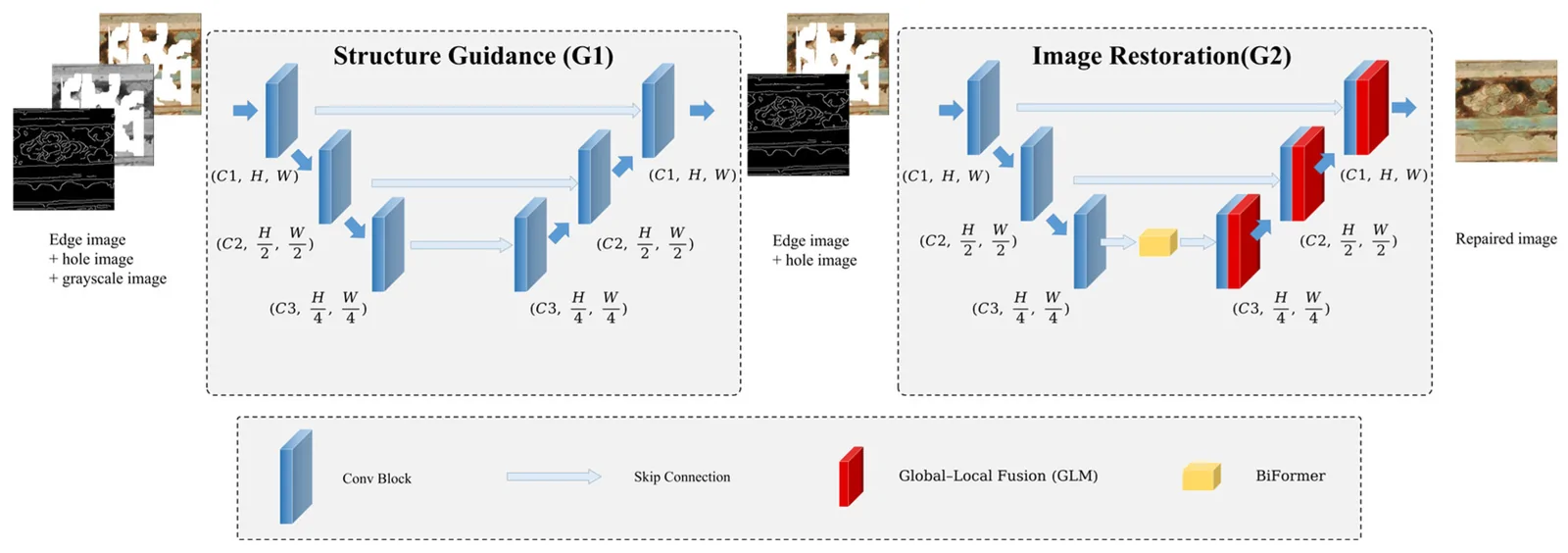
Overall architecture of proposed MuralsNet with BiFormer and GLM.

Although explicit edge guidance provides useful structural constraints, the original EdgeConnect framework remains limited in modeling long-range relationships among spatially separated decorative elements and in coordinating semantic information with local details during decoding. These limitations become more evident in regions containing repeated motifs, symmetric structures, and complex pattern boundaries. Therefore, MuralsNet modifies the G2 image completion network at two feature-processing stages, as shown in Figure 2. BiFormer is introduced into the bottleneck, where the compressed feature representation is used to model long-range structural dependencies. GLM is placed in the decoder to enhance the interaction between high-level semantic information and local spatial details during feature reconstruction. With BiFormer and GLM operating at different stages of G2, the network incorporates global structural modeling and local feature refinement into the EdgeConnect-based image completion process.

### 3.2. BiFormer Module

In MuralsNet, global structural reasoning is implemented using the BiFormer module. For Dunhuang decorative mural restoration, the missing regions often contain incomplete decorative structures, such as repeated motifs, symmetric patterns, and continuous contour lines. These structures cannot be fully recovered only from adjacent pixels because the corresponding references may exist in distant regions of the mural image. Therefore, establishing long-range structural dependencies is essential for restoring the global arrangement and semantic consistency of decorative patterns.

To achieve this goal, BiFormer is introduced into the bottleneck stage of the G2 U-Net. Transformer-based restoration architectures, such as Uformer [23] and Restormer [24], demonstrate the effectiveness of hierarchical attention mechanisms for high-resolution image restoration. In MuralsNet, the bottleneck position is selected because the feature maps have been compressed through downsampling operations, where high-level semantic information is preserved while detailed textures are reduced. This makes the bottleneck feature representation suitable for modeling global structural relationships rather than local texture reconstruction.

BiFormer adopts Bi-level Routing Attention [25], which dynamically selects windows related to the target region and establishes long-range dependencies while maintaining relatively low computational complexity. Accordingly, BiFormer is suitable for repeated patterns and cross-region structure modeling. Compared with conventional Transformers, BiFormer does not compute global attention over all windows, but only focuses on regions related to the target pattern, making it more suitable for modeling recurring decorative patterns commonly observed in Dunhuang murals. Compared with conventional global attention mechanisms such as Vision Transformer [26] and shifted-window attention strategies such as Swin Transformer and SwinIR [27,28], BiFormer reduces unnecessary interactions among irrelevant windows by dynamically selecting task-relevant windows. These properties make it potentially suitable for modeling repeated patterns, directional lines, and complex boundaries in Dunhuang decorative murals.

**Figure 3:**
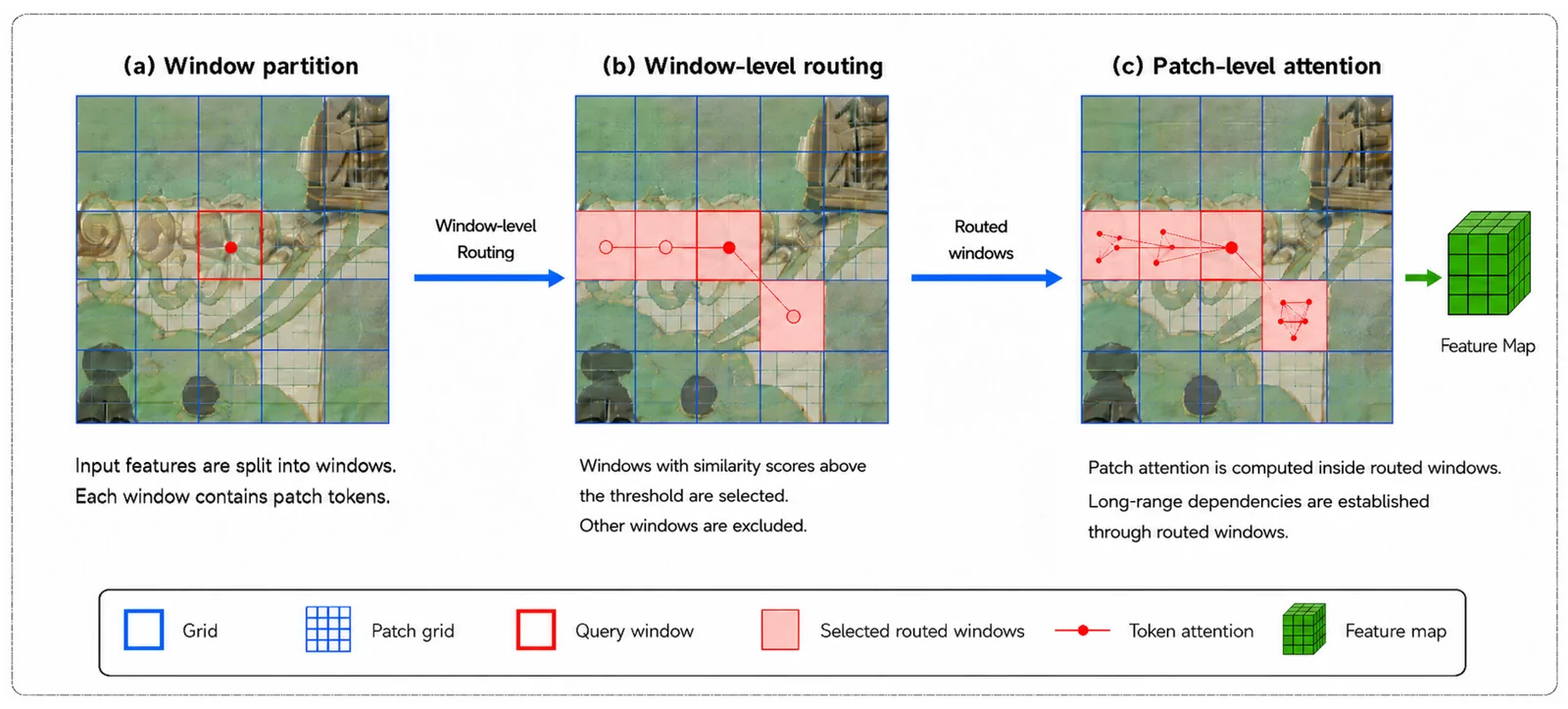
Structure of Bi-level Routing Attention module.

Specifically, BiFormer consists of Layer Normalization, Bi-level Routing Attention, and an MLP, as shown in Figure 3. Since Dunhuang decorative patterns contain repeated units and cross-region correspondences, the routing mechanism can preferentially focus on windows with similar patterns to the missing region.

The processing of the Bi-level Routing Attention module mainly consists of two stages. In the first stage, the module computes global correlations across all windows by measuring the similarity between each window and the target region window, selecting only the windows with high relevance.

Let the input feature map be X, which is divided into $N_{w}$ windows to obtain $X_{w}$, where P denotes the number of patches per window, and C denotes the channel dimension. The definition of inter-window similarity is shown in Equation (1).

(1) $$S_{ij}=\frac{Q_{i}\cdot K_{j}}{\left||Q_{i}||\cdot ||K_{j}|\right|},\;i,j=1,\ldots ,N_{w},$$

where $Q_{i}$ and $K_{j}$ represent the query and key vectors of window i and window j, respectively. Cosine similarity is used to measure the relevance between windows. In the implementation adopted in this study, window routing is determined according to the inter-window similarity, with the routing threshold τ set to 0.7 in this study. Windows satisfying $S_{ij}>\tau$ are retained for subsequent fine-grained attention computation; information from other windows is suppressed or ignored. This strategy ensures that the network focuses only on windows highly relevant to the target region at the global scale, effectively reducing interference from irrelevant information and improving computational efficiency.

In the second stage, fine-grained self-attention computation is performed among the patches within the retained windows to enhance local feature modeling capabilities. Let the retained window set be denoted as ${\hat{X}}_{w}$, and the attention weights between patches within the window are calculated as shown in Equation (2).

(2) $$A_{pq}=\frac{\exp\left(\frac{Q_{p}K_{q}^{T}}{\sqrt{d}}\right)}{\sum_{r=1}^{P}\exp\left(\frac{Q_{p}K_{r}^{T}}{\sqrt{d}}\right)}p,q=1,\ldots ,P,$$

where d denotes the dimensionality of the feature vectors, and $Q_{p}$ and $K_{q}^{T}$ represent the query vector of patch p and the key vector of patch q, respectively. The value vectors $V_{j}$ are aggregated through a weighted summation operation to obtain the final feature representation of the patches within each window, as defined in Equation (3).

(3) $$Y_{i}=\sum_{j=1}^{P}A_{ij}V_{j},$$

Here, $Y_{i}$ denotes the final output feature representation within window i, namely the feature vector obtained after aggregating relevant patch information through the bi-level routing attention mechanism, and $A_{ij}$ represents the contribution weight. Through this mechanism, the module combines global window selection with local patch-level refinement, preserving relevant global contextual information while maintaining fine-grained modeling of local textures and details.

After Bi-level Routing Attention computation, the resulting features are further processed with layer normalization, residual connections, and a multi-layer perceptron to form the complete BiFormer module. In this study, BiFormer is embedded into the bottleneck stage of G2 U-Net to introduce long-range contextual interactions into the low-resolution semantic feature space, with particular emphasis on repeated patterns, directional structures, and complex boundaries in Dunhuang decorative mural images.

### 3.3. Global–Local Fusion Module (GLM)

In MuralsNet, local refinement is implemented by the proposed GLM. During the decoder stage of G2 U-Net, feature reconstruction requires the effective integration of high-level semantic representations from decoder layers and spatial details from encoder skip connections. The original U-Net mainly fuses these features through skip connections followed by convolution. Although this strategy preserves information from different feature levels, it may be insufficient for Dunhuang decorative mural images with complex textures and structural patterns, particularly in regions containing intricate boundaries and fine decorative details. As a result, semantic information and local texture features may not be sufficiently coordinated during feature reconstruction, leading to boundary discontinuities, texture inconsistency, and inaccurate color transitions in complex decorative mural regions.

**Figure 4:**
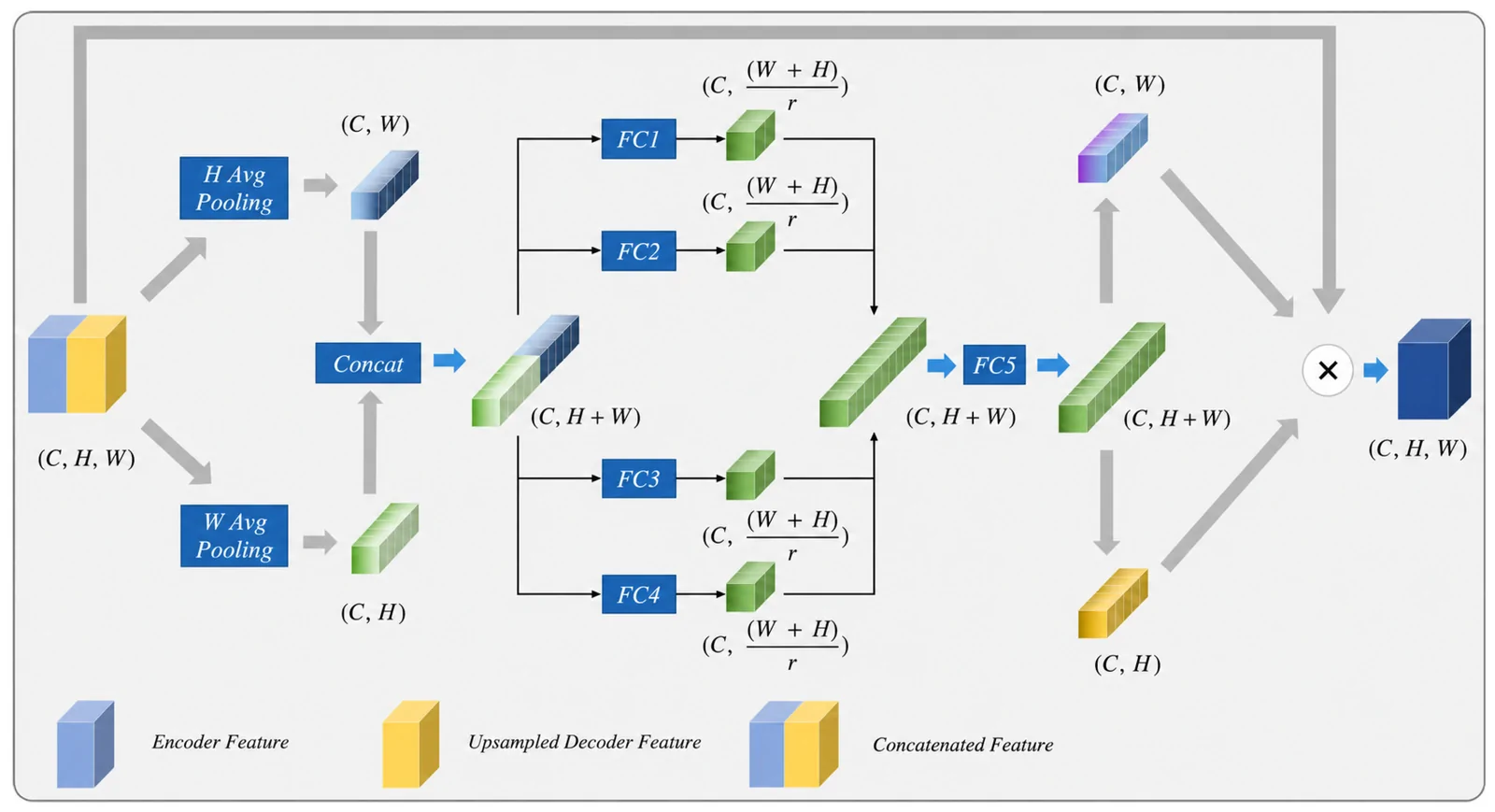
Structure of proposed Global–Local Fusion Module.

To improve feature fusion during decoding, a Global–Local Fusion Module (GLM) is introduced into G2 U-Net. GLM uses high-level semantic information to guide local feature reconstruction while preserving useful texture and detail information from the encoder. As shown in Figure 4, GLM consists of two main components: coordinate decomposition and widened channel attention. The coordinate decomposition component encodes directional positional information to enhance spatial feature representation, while the widened channel attention component strengthens feature interaction in the channel dimension. Through the combination of these two components, GLM refines the fused features from both spatial and channel perspectives, facilitating the reconstruction of mural textures and local structural details.

In the decoder stage of G2 U-Net, the upsampled features from the previous decoder layer contain rich semantic information, while the output of the corresponding encoder layer preserves more precise local spatial information. These two sets of features are concatenated along the channel dimension and fed into the GLM as fused input features, providing the basis for subsequent information aggregation and weight assignment.

Recent edge-aware image restoration methods have demonstrated that explicit modeling of structural boundaries is beneficial for preserving fine details and edge continuity [29]. In the coordinate decomposition component, the GLM draws on the directional positional encoding strategy of Coordinate Attention [30]. Instead of compressing spatial information into a single global descriptor, feature information is aggregated separately along the horizontal and vertical directions, allowing positional information in both directions to be retained. Specifically, for the input feature tensor $X\in R^{C\times H\times W}$, global average pooling is performed separately along the horizontal and vertical directions. Two pooling kernels of size (H,1) and (1,W) are used to independently encode each channel along the vertical and horizontal directions, respectively, thereby preserving directional positional information in the resulting feature representations.

At a given height h, the output value of the c-th channel can be calculated using the following formula, as shown in Equation (4).

(4) $$z_{c}\left(h\right)=\frac{1}{W}\sum_{i=1}^{W}x_{c}\left(h,\;i\right)$$

Similarly, at a given width w, the output of the c-th channel is calculated as shown in Equation (5).

(5) $$z_{c}\left(w\right)=\frac{1}{H}\sum_{j=1}^{H}x_{c}\left(j,\;w\right)$$

The two directional encoding operations generate feature representations along the horizontal and vertical directions, respectively. Unlike conventional channel attention, which compresses spatial information into a single global descriptor, directional encoding preserves spatial information along one direction while capturing long-range contextual information along the other. In implementation, the two one-dimensional feature representations are subsequently concatenated along the channel dimension and passed through a shared 1×1 convolution to fuse the directional information. The fused feature is then processed by two independent 1×1 convolutional layers to generate the horizontal and vertical attention maps, respectively. A sigmoid activation function is applied to obtain normalized directional attention weights, which are subsequently multiplied element-wise with the input features to perform direction-aware spatial feature enhancement.

On the basis of the direction-aware feature representation, GLM further introduces a widened channel attention component to enhance feature interaction across different feature subspaces. Specifically, four parallel branches, denoted as FC1–FC4 in Figure 4, are constructed. Each branch contains an independent fully connected layer and performs dimensionality reduction on the input feature representation. Through the four parallel transformation paths, different channel-dependent feature representations can be learned simultaneously rather than relying on a single transformation path.

The outputs of the four branches are subsequently concatenated and passed through an integration layer to aggregate the information learned from the different feature subspaces. The integrated representation is then used to generate adaptive feature weights. Finally, the generated weighted feature representation is multiplied with the input feature to obtain the output of the GLM.

Through directional positional encoding and widened multi-branch channel interaction, the GLM enhances the interaction between semantic information and local detail features during decoding. The module therefore serves as the local feature refinement component of MuralsNet, supporting the reconstruction of texture transitions, color distributions, and local structural details in damaged mural regions.

### 3.4. Loss Function

Based on the two-stage EdgeConnect framework, this study introduces structural improvements while retaining the original training strategy for the loss function [9]. The model training consists of an edge generation stage and an image inpainting stage, with each stage, respectively, constraining structure prediction and image content generation.

Let the original complete image be I, and the defect mask be M, where M = 1 represents the defective region, M = 0 represents the known region, and ⊙ represents element-wise multiplication. Then the input damaged image can be represented as shown in Equation (6):

(6) $$I_{in}\;=I⊙\left(1-M\right)$$

In the edge generation stage, the network predicts the edge structure of the missing region based on the damaged image and the mask. Let the predicted edge map be $E_{pred}$ and the real edge map be $E_{gt}$. The loss in this stage consists of edge reconstruction loss and edge adversarial loss, as shown in Equation (7):

(7) $$L_{edge}=\lambda_{er}\;L_{er}\;+\lambda_{ea}\;L_{ea}$$

Among them, the edge reconstruction loss $L_{er}$ uses $L_{1}$ distance constraint to minimize the difference between the edge and the real edge, as shown in Equation (8):

(8) $$L_{er}\;={∥E_{pred}\;-E_{gt}∥}_{1}$$

The edge adversarial loss $L_{ea}$ constrains the authenticity of generated edges through an edge discriminator, making the predicted edges closer to the real edges in terms of spatial location, structural morphology, and overall distribution, thus providing a reliable structural prior for subsequent image inpainting.

In the image inpainting stage, the inpainting network takes the damaged image, mask, and predicted edge map as input to generate the final inpainted image $I_{pred}$. The loss in this stage consists of pixel reconstruction loss, perceptual loss, style loss, and image adversarial loss, as shown in Equation (9):

(9) $$L_{img}\;=\lambda_{r}{\;L}_{r}\;+\lambda_{p}L_{p}\;+\lambda_{s}\;L_{s}s+\lambda_{a}{\;L}_{a}a$$

$L_{r}$ uses $L_{1}$ distance constraints to ensure the consistency between the generated image and the real image at the pixel level, as shown in Equation (10):

(10) $$L_{r}\;={∥I_{pred}\;-I∥}_{1}$$

Perceptual loss [31] constrains semantic consistency through high-level features of the pre-trained network, style loss constrains texture and style distribution through Gram matrix [32], and image adversarial loss is used to improve the visual realism of the restoration result. The final overall loss is shown in Equation (11).

(11) $$L_{total}=L_{edge}+L_{img}$$

These losses provide optimization constraints for structure prediction and image reconstruction, enabling structure guidance, global reasoning, and local refinement to be jointly trained under a unified optimization objective.

## 4. Experimental Analysis

### 4.1. Experimental Setup

All experiments are conducted on a self-constructed Dunhuang decorative mural image dataset, as described in detail in the previous section. To ensure fair and comparable results, all experiments are performed on an NVIDIA RTX 4090D GPU, and all compared methods are trained and tested under the same data splits and unified training settings.

For model training, all input images are resized to 256 × 256 pixels, with a batch size of 16. The Adam optimizer is adopted with $\beta_{1}=0.1$ and $\beta_{2}=0.9$. The learning rates of both the generator and discriminator are set to 1 × 10^−4^, and the network is trained for approximately 93,000 iterations.

For quantitative evaluation, the Peak Signal-to-Noise Ratio (PSNR), the Structural Similarity Index Measure (SSIM) [33], Mean Absolute Error (MAE), Learned Perceptual Image Patch Similarity (LPIPS) [34], and Masked Edge F1 are employed. The PSNR and SSIM measure the pixel-level fidelity and structural similarity between the inpainted images and the ground truth, while MAE reflects the overall reconstruction error of the images. LPIPS is used to evaluate perceptual similarity, where a lower value indicates better perceptual consistency. To further evaluate structural preservation within the damaged regions, Masked Edge F1 is introduced. For Masked Edge F1, edge maps are extracted from the restored and ground-truth images using the Canny detector with thresholds of 100 and 200. The binary damage mask is dilated outward by 2 pixels to include the local boundary neighborhood, and edge matching is performed within this region with a spatial tolerance of 2 pixels. Precision and recall are calculated based on the matched predicted and ground-truth edge pixels, respectively, and their harmonic mean is reported as the Masked Edge F1 score. A higher value indicates better preservation of local edge and contour structures. All reported quantitative results are averaged over the complete test set of 5589 images.

The computational efficiency of MuralsNet is evaluated by comparing it with the EdgeConnect baseline in terms of the number of parameters, FLOPs, average inference time, and peak training GPU memory. All measurements were conducted under the same hardware environment and experimental settings with an input resolution of 256 × 256, and the parameter and FLOP calculations cover the complete two-stage pipeline. As shown in Table 1, MuralsNet contains 22.71 M parameters and requires 136.49 G FLOPs, compared with 21.54 M parameters and 122.64 G FLOPs for EdgeConnect, corresponding to increases of 5.43% and 11.29%, respectively. The average inference time increases from 7.10 to 11.49 ms per image, while the peak training GPU memory increases from 5.96 to 9.01 GB. These results show that the introduction of BiFormer and the GLM increases the computational and memory requirements, with the increase being more pronounced in actual inference time and training memory than in model size and theoretical computational complexity.

### 4.2. Comparative Experiment

To verify the overall performance of the proposed method in the Dunhuang mural image restoration task, five representative image inpainting methods, DeepFillv2 [35], GLNet [36], PCNet [37], RePaint [38], and LaMa [15], are selected for comparison. All methods are trained and tested on the same dataset and under unified experimental settings. The experimental results are shown in Table 2.

As shown in Table 2, MuralsNet achieves the highest PSNR and SSIM among the evaluated methods, reaching 23.59 dB and 0.9669, respectively. It also obtains the lowest LPIPS of 0.0724 and the highest Masked Edge F1 of 0.8948, indicating better perceptual similarity and stronger preservation of edge and contour structures within the damaged regions. LaMa achieves the lowest MAE of 0.0403, while its PSNR, SSIM, LPIPS, and Masked Edge F1 are 22.50 dB, 0.6913, 0.0879, and 0.8830, respectively. These results indicate that different evaluation metrics reflect different aspects of the restoration performance.

DeepFillv2, GLNet, and PCNet show less favorable quantitative performance on most metrics, indicating greater reconstruction errors, lower perceptual similarity, or weaker structural preservation under the evaluated irregular-mask setting. Among the comparison methods, RePaint shows relatively strong structural similarity, with an SSIM of 0.9533, while its LPIPS and Masked Edge F1 are 0.0806 and 0.8757, respectively. However, visually plausible generation does not necessarily correspond to higher reference-based similarity. Diffusion-based inpainting may generate reasonable textures or structures that differ from the original content within the masked region, resulting in lower reference-based similarity even when the restored image appears visually natural.

**Figure 5:**
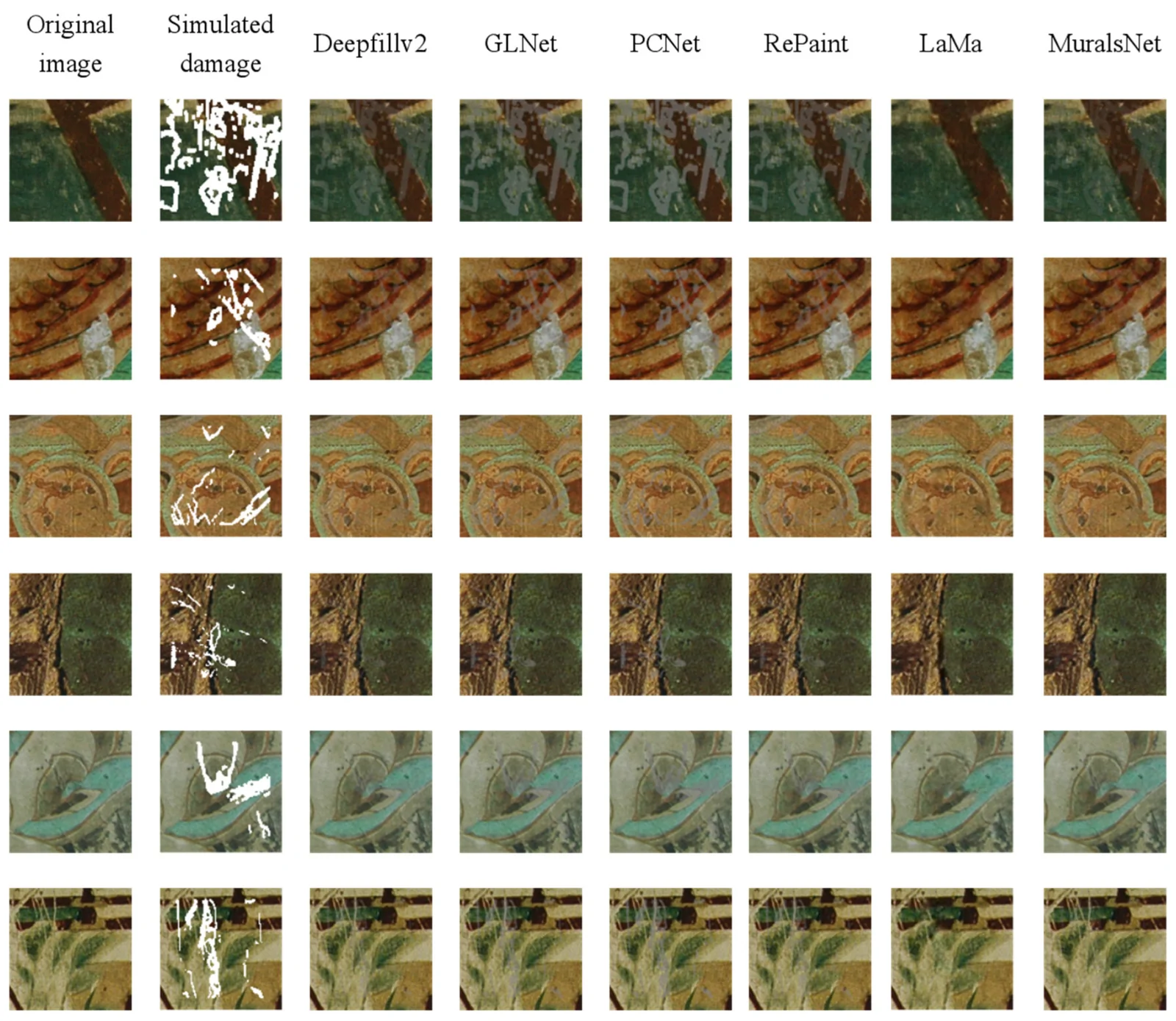
Visual comparison of different image inpainting methods.

The comparison results of different image inpainting methods on the Dunhuang decorative mural dataset are shown in Figure 5. In low-frequency regions such as color-block backgrounds, DeepFillv2 and RePaint can both complete basic content filling, but their performance decreases in pattern-dense regions. DeepFillv2 produces relatively smooth textures in some examples, whereas RePaint generates visually natural content but shows differences from the original patterns in some detailed regions. GLNet preserves the overall structure in several cases, although local details around complex boundaries remain less consistent with the ground truth. PCNet also reconstructs the missing regions, but differences in local patterns and color transitions can still be observed. LaMa performs well in completing relatively large missing regions and produces visually coherent filled areas. However, its results tend to be over-smoothed in regions containing fine decorative lines, where thin contours may become blurred, discontinuous, or less visible after restoration. In comparison, MuralsNet produces results that are visually closer to the ground truth in several pattern-dense regions, particularly in the reconstruction of decorative contours and local texture details. These qualitative observations are consistent with the lower LPIPS and higher Masked Edge F1 obtained by MuralsNet.

### 4.3. Ablation Experiment

To verify the effectiveness of the BiFormer module and the GLM in the restoration of Dunhuang decorative mural images, ablation experiments are conducted based on EdgeConnect. The experiments analyze the independent contribution of each module to the model performance by progressively introducing different modules. The model’s performance is evaluated using the PSNR, the SSIM, MAE, LPIPS, and Masked Edge F1, as shown in Table 3.

Table 3 further shows the quantitative effects of introducing each module. When neither BiFormer nor GLM is introduced, the baseline EdgeConnect model achieves a PSNR of 23.27 dB, an SSIM of 0.9643, an MAE of 0.0696, an LPIPS of 0.1031, and a Masked Edge F1 of 0.8322. After introducing BiFormer, the PSNR and SSIM increase to 23.42 dB and 0.9659, respectively, while the MAE decreases slightly to 0.0693. Meanwhile, the LPIPS decreases to 0.1007 and the Masked Edge F1 increases to 0.8571, indicating improvements in perceptual similarity and local edge preservation. These results suggest that BiFormer contributes to the reconstruction of structurally relevant information by modeling long-range dependencies.

When the GLM is introduced individually, the PSNR increases to 23.48 dB and the MAE decreases to 0.0673, while the SSIM remains close to that of the baseline at 0.9644. The LPIPS is further reduced to 0.0900, and the Masked Edge F1 increases to 0.8669. This indicates that the GLM contributes not only to reducing reconstruction errors but also to improving perceptual similarity and contour preservation through global–local feature fusion. When BiFormer and the GLM are jointly incorporated, MuralsNet achieves the best overall performance among the four configurations, with a PSNR of 23.59 dB, an SSIM of 0.9669, an MAE of 0.0652, an LPIPS of 0.0724, and a Masked Edge F1 of 0.8948. These results indicate that BiFormer and the GLM provide complementary contributions to reconstruction fidelity, perceptual similarity, and local structural preservation.

The uncertainty of these performance differences was evaluated using a paired bootstrap analysis over the 5589 test images. The mean differences (MuralsNet − EdgeConnect) and the corresponding 95% confidence intervals were +0.3179 [0.2802, 0.3556] for PSNR, +0.0026 [0.0025, 0.0027] for SSIM, −0.0046 [−0.0049, −0.0043] for MAE, −0.0306 [−0.0327, −0.0285] for LPIPS, and +0.0625 [0.0614, 0.0636] for Masked Edge F1. None of the confidence intervals included zero, indicating that the observed improvements were consistent under paired resampling of the test set.

**Figure 6:**
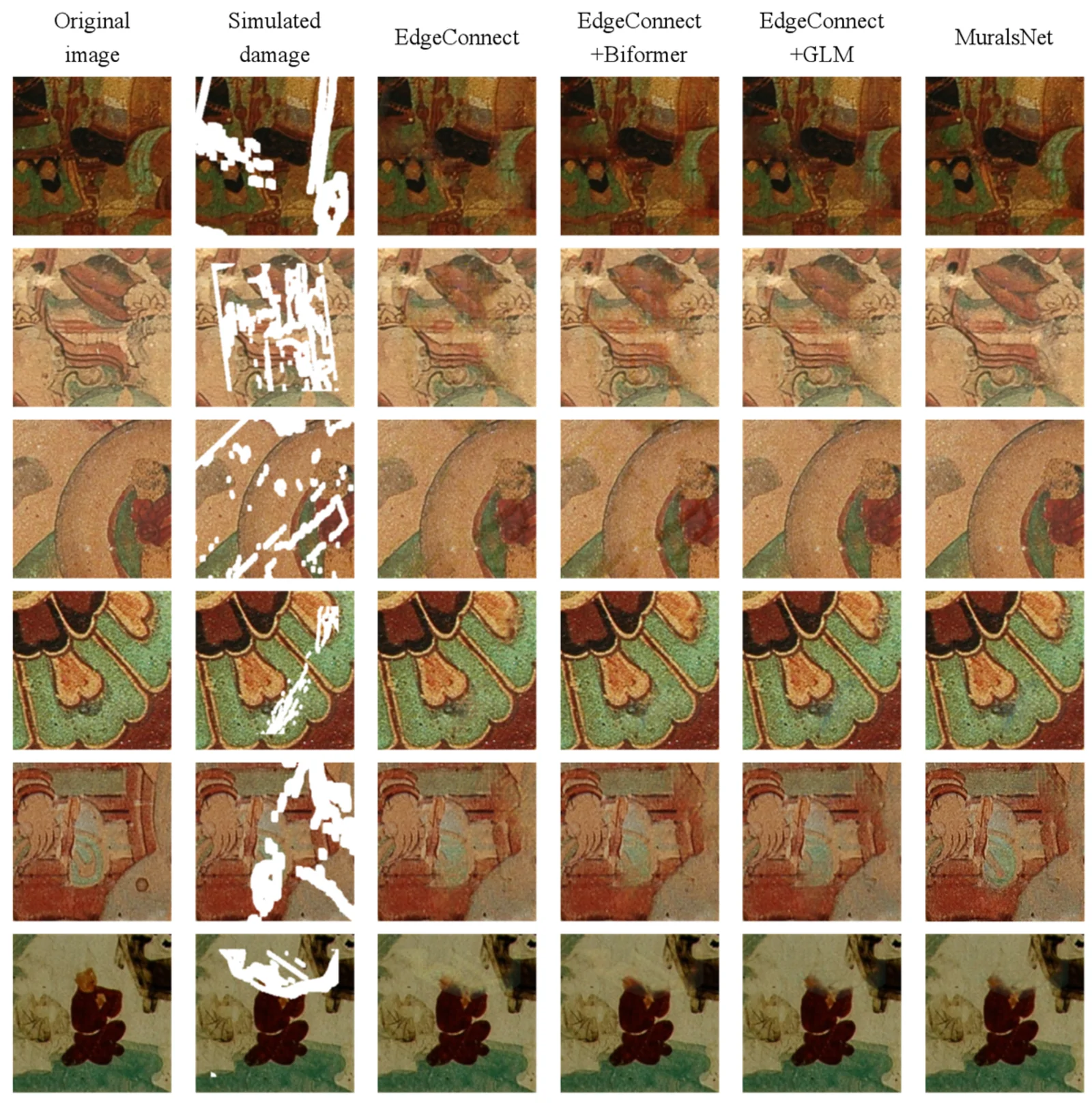
Visual comparison of different configurations in the ablation experiment.

To further examine the individual and combined effects of BiFormer and GLM, Figure 6 presents the visual results of the EdgeConnect baseline, the two single-module variants, and the complete MuralsNet. Compared with the baseline, the BiFormer variant shows more coherent reconstruction in some regions containing repeated patterns and spatially separated decorative structures, while the GLM variant shows improved local texture transitions and boundary reconstruction in several examples. When BiFormer and the GLM are introduced together, MuralsNet further improves the overall consistency of the restored regions in several examples, with more coherent decorative structures and local details. These visual observations are consistent with the quantitative trends in Table 3, indicating that BiFormer and the GLM provide complementary improvements when integrated into the complete framework.

**Figure 7:**
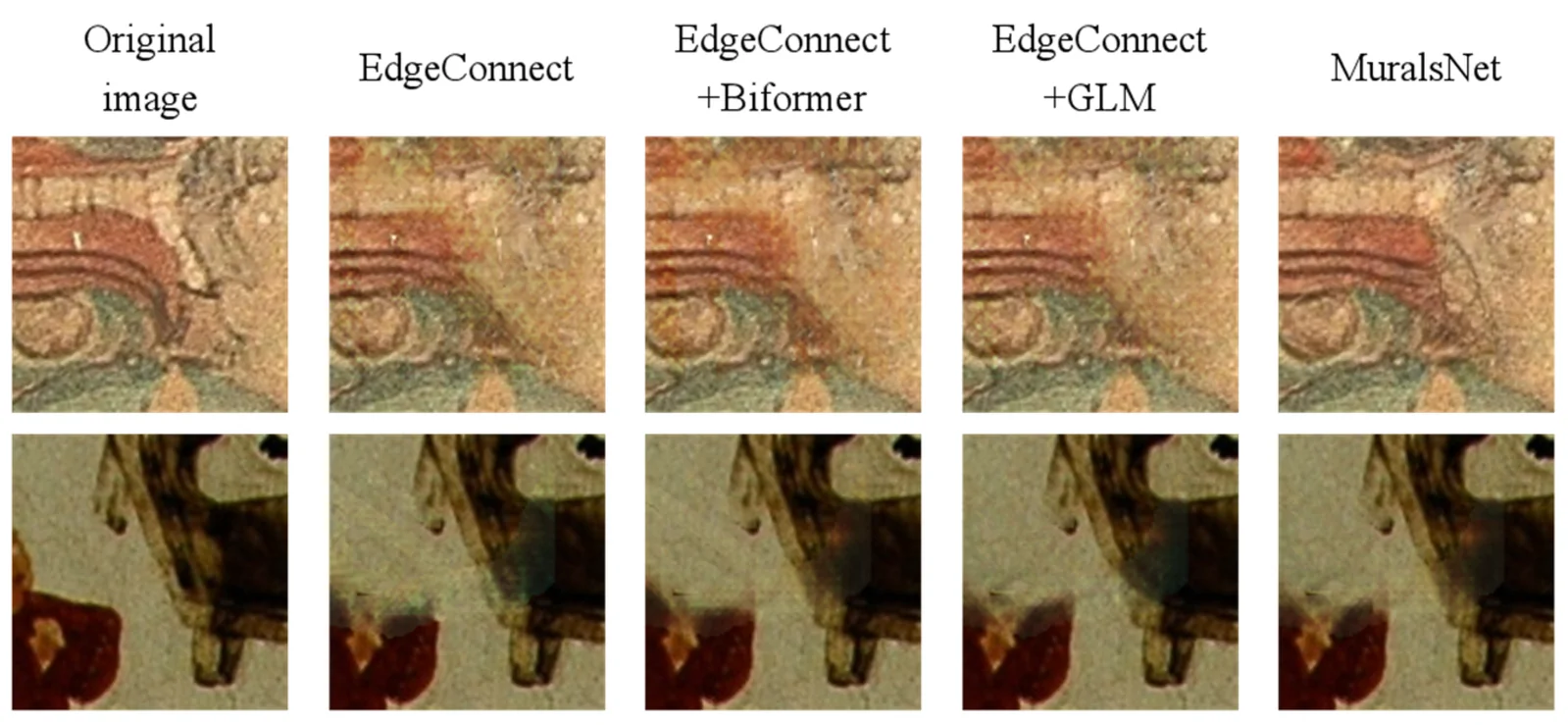
Enlarged local comparison of the ablation experiments.

Figure 7 provides enlarged views of representative regions from Figure 6 for a closer comparison of the local restoration details. Compared with the EdgeConnect baseline, the variants incorporating BiFormer and the GLM show improved preservation of local boundaries and fine structures to different degrees. The complete MuralsNet provides a more balanced restoration of structural and appearance details in these regions.

## 5. Conclusions

In this study, two targeted modifications are introduced for Dunhuang decorative mural image inpainting. First, the BiFormer module based on a bi-level attention mechanism is integrated into the bottleneck layer of G2 U-Net to enhance global semantic modeling and improve structural consistency in missing regions. Second, a Global–Local Fusion Module (GLM) is designed in the decoder stage to facilitate effective fusion between local encoder features and upsampled decoder features, strengthening local detail restoration. The restoration performance is comprehensively evaluated using PSNR, SSIM, MAE, LPIPS, and Masked Edge F1. Comparative experiments show that MuralsNet achieves the best PSNR, SSIM, LPIPS, and Masked Edge F1 among the evaluated methods, while LaMa obtains the lowest MAE. Ablation experiments further demonstrate that BiFormer and GLM provide complementary improvements in reconstruction fidelity, perceptual similarity, and local structural preservation. Visual comparisons provide qualitative observations consistent with these quantitative results, particularly in the preservation of decorative contours and local structural details.

Although MuralsNet achieves competitive performance, several limitations remain. First, for severely degraded mural regions with very limited surrounding contextual information, the reconstruction of highly detailed textures may remain challenging. Second, the current model is trained and evaluated primarily on 256 × 256 image patches with simulated mask-based damage. Although this setting enables controlled quantitative evaluation using the original images as ground truth, real mural deterioration may involve more complex degradation patterns, such as pigment fading, uneven surface contamination, and layered material loss. In addition, as MuralsNet retains the two-stage EdgeConnect framework, inaccuracies in the edge prediction stage may propagate to the subsequent image completion stage and affect the final reconstruction. Furthermore, although LPIPS and Masked Edge F1 provide complementary evaluations of perceptual similarity and local structural preservation, the current evaluation does not fully capture all aspects of visual quality and structural fidelity in complex real-world mural degradation. Therefore, the current results should be regarded as digital reconstruction under the available visual evidence rather than a definitive recovery of historically authentic content. Future work will incorporate real damaged mural samples, investigate restoration at higher resolutions, and explore more comprehensive evaluation protocols and robust multi-modal priors to improve the applicability of the method in practical cultural heritage restoration scenarios. Taken together, this work provides a practical global–local collaborative restoration framework for Dunhuang decorative murals and offers a reference for image restoration in broader cultural heritage scenarios.

## Figures and Tables

**Table 1:** Comparison of Computational Efficiency between EdgeConnect and MuralsNet.

Method	Params (M) ↓	FLOPs (G) ↓	Avg. Inference Time (ms/Image) ↓	Peak Training GPU Memory (GB) ↓
EdgeConnect	21.54	122.64	7.10	5.96
MuralsNet	22.71	136.49	11.49	9.01

Note: ↓ indicates lower values are better.

**Table 2:** Quantitative comparison of different image inpainting methods on the Dunhuang mural dataset.

Method	PSNR	SSIM	MAE	LPIPS	Masked Edge F1
DeepFillv2	21.30	0.7352	0.0722	0.0943	0.8746
GLNet	22.79	0.8379	0.0745	0.1078	0.8612
PCNet	20.18	0.7051	0.0825	0.1329	0.8467
RePaint	22.57	0.9533	0.0705	0.0806	0.8757
LaMa	22.50	0.6913	**0.0403**	0.0879	0.8830
MuralsNet	**23.59**	**0.9669**	0.0652	**0.0** **724**	**0.** **8948**

Note: Bold values indicate the best performance for each metric.

**Table 3:** Quantitative results of ablation experiments on BiFormer and GLM.

BiFormer	GLM	PSNR	SSIM	MAE	LPIPS	Masked Edge F1
×	×	23.27	0.9643	0.0696	0.1031	0.8322
√	×	23.42	0.9659	0.0693	0.1007	0.8571
×	√	23.48	0.9644	0.0673	0.0900	0.8669
√	√	23.59	0.9669	0.0652	0.0724	0.8948

Note: “√” and “×” indicate the presence and absence of the corresponding module, respectively.

## Data Availability

The original mural images used in this study were obtained from published cultural heritage resources. Due to copyright restrictions and licensing limitations, the processed dataset cannot be publicly released. However, the data processing pipeline and experimental settings are described in detail to facilitate reproduction.
